# Comparative outcomes of bioabsorbable poly-4-hydroxybutyrate versus synthetic mesh in ventral hernia repair: A propensity-matched cohort study

**DOI:** 10.1007/s10029-026-03848-8

**Published:** 2026-09-16

**Authors:** Kartik S. Akkihal, Kelly M. Dickerson, Anya Ittiruck, Gerald O. Ogola, Christine Wang, Mazen Iskander, Steven G. Leeds, Marc A. Ward, Bola Aladegbami

**Affiliations:** 1 Department of Minimally Invasive Surgery, Baylor University Medical Center, 3500 Gaston Ave, Dallas, TX 75246 USA; 2 Texas A&M College of Medicine at Baylor University Medical Center, Dallas, TX USA; 3 Research Institute, Baylor Scott and White Health, Dallas, TX USA; 4 Bariatric Surgery, Baylor Scott and White, Waxahachie, TX USA

**Keywords:** Ventral hernia repair, Poly‑4‑hydroxybutyrate mesh, Biosynthetic mesh, Propensity score matching, Surgical site infection, Hernia recurrence

## Abstract

**Purpose:**

Poly-4-hydroxybutyrate (P4HB) biosynthetic mesh offers a bioabsorbable alternative to permanent synthetic mesh in ventral hernia repair, but comparative outcome data are limited by selection bias, as P4HB is preferentially used in complex and contaminated cases.

**Methods:**

We performed a retrospective cohort study of adults undergoing ventral hernia repair with P4HB, biologic, or permanent synthetic mesh at a single tertiary center from January 2014 to January 2025. The primary outcome was hernia recurrence. Secondary outcomes included 30-day postoperative complications, mesh infection, and time to recurrence. Of 506 patients, 32 with non-ventral hernias and 71 with missing mesh type data were excluded, leaving 403 ventral hernia repairs for analysis (45 P4HB, 22 biologic, and 336 permanent synthetic). Patients receiving P4HB were matched 1:1 to patients receiving permanent synthetic mesh using propensity scores derived from age, sex, body mass index, diabetes, CDC wound class, Ventral Hernia Working Group grade, and hernia size.

**Results:**

In the unmatched cohort, P4HB patients had larger hernia defects, higher VHWG grades, more contaminated CDC wound classes, and longer operative times than patients receiving permanent synthetic mesh. Mesh infection and hernia recurrence were low and did not differ significantly between groups, but P4HB patients had higher rates of 30-day reoperation, ileus, and small bowel obstruction. After propensity score matching (25 P4HB vs 25 permanent synthetic), operative time was similar between groups. Hernia recurrence remained uncommon and was not significantly different (4.0% vs 8.0%, *p*=0.55), and mesh infection rates were also low (0% vs 8.0%, *p*=0.15). P4HB was associated with lower 30-day surgical site infection (0% vs 16.7%, *p*=0.04) and surgical site occurrence (4.2% vs 33.3%, *p*=0.01), while other 30-day outcomes were not significantly different.

**Conclusions:**

In this retrospective, single-center propensity-matched cohort, long-acting resorbable poly-4-hydroxybutyrate mesh demonstrates comparable recurrence and mesh infection rates with lower short-term wound morbidity after balancing key risk factors, suggesting bioabsorbable mesh to be a reasonable alternative to synthetic mesh in selected complex or contaminated ventral hernia repairs. However, the modest matched sample size, exclusions due to missing data, and follow-up shorter than the expected P4HB resorption period warrant cautious interpretation and emphasize the need for larger studies with longer-term follow-up.

**Supplementary Information:**

The online version contains supplementary material available at https://doi.org/10.1007/s10029-026-03848-8.

## Introduction

Ventral hernias are common with more than 400,000 repairs being performed annually in the United States, and permanent synthetic mesh remains the standard of care to prevent hernia recurrence [1, 2]. While inexpensive and durable, permanent synthetic mesh is associated with a broad complication profile, including mesh infection sometimes requiring mesh explantation, and enterocutaneous fistula formation [3]. To combat the increased risk of mesh infection in high-risk repairs, biologic mesh was developed, but randomized trials have demonstrated significantly higher recurrence rates with approximately 20-fold higher cost compared to synthetic mesh, and no actual reduction in infection complications [4, 5].

Poly-4-hydroxybutyrate (P4HB, Phasix) is a long-acting bioabsorbable mesh designed to provide temporary mechanical support while gradually resorbing over approximately 12 to 18 months, with the goal of minimizing chronic mesh-related complications, including chronic pain and mesh allodynia that can occur with permanent synthetic mesh [6]. Although our cohort has a mean follow-up of approximately 12 months, longer-term series of P4HB mesh have specifically highlighted its potential to reduce chronic mesh pain and allodynia, which remain important considerations for complex ventral hernia repair. Data supporting P4HB use has been variable with reported recurrence rates ranging from 9% to 22% at long-term follow-up and various complication profiles across different patient populations and wound classifications [7, 8]. These studies have been limited by sample size, heterogeneous populations, and the absence of well-matched comparisons between permanent synthetic mesh. Clinical decision making is further clouded by emerging evidence showing that biosynthetic meshes may be preferentially selected for more challenging cases with higher Ventral Hernia Working Group (VHWG) grades and greater wound contamination [9].

At our institution and throughout published literature, P4HB mesh appears to be utilized more frequently in complex cases with higher VHWG grades and higher Center for Disease Control (CDC) wound classifications, creating inherent selection bias that confounds direct outcome comparisons with synthetic mesh. The goal of our study was to address this selection bias by using propensity score matching to balance key clinical variables between P4HB and synthetic mesh cohorts, with the purpose of providing a more accurate estimate of the relative performance of these two mesh types in terms of hernia recurrence and short-term postoperative outcomes.

## Methods

This was a retrospective cohort study using a prospectively maintained database of patients undergoing ventral hernia repair at a single tertiary-care center from January 2014 to January 2025. Patients were included if they were aged 18 years or older and underwent ventral hernia repair with synthetic, biologic, or P4HB mesh. Patients were excluded if they underwent non-ventral hernia repair, were younger than 18 years, or had missing key perioperative variables required for analysis.

Demographic and clinical variables were extracted. Comorbidities included diabetes mellitus, hypertension, chronic obstructive pulmonary disease, obstructive sleep apnea, inflammatory bowel disease, cancer history, end-stage renal disease, and prior thrombosis. Hernia characteristics included defect size, mesh size, complexity characterized by VHWG grade [10], and wound contamination categorized by CDC wound class [11]. Operative variables included operative time and mesh type, while postoperative variables included mesh infection and hernia recurrence. Hernia defect dimensions were measured intraoperatively with a ruler and area was calculated in square centimeters from the recorded length and width in the operative note. Thirty‑day postoperative outcomes included surgical site infection (SSI), surgical site occurrence (SSO), reoperation, ileus, small bowel obstruction, urinary tract infection, renal failure, pulmonary embolism, deep venous thrombosis, pneumonia, respiratory failure, unplanned intensive care unit transfer, mortality, other complications, and readmission. SSO was defined as a wound-related postoperative event, including seroma, hematoma, wound dehiscence, fat necrosis, enterocutaneous fistula, or SSI, defined as infections of the incision, organ, or space occurring after surgery [12, 13]. Hernia recurrence was defined as a recurrent fascial defect identified on physical examination. Follow-up duration and timing of adverse events were obtained from clinic notes, operative reports, imaging, and readmission records. Time to recurrence was recorded in months from index repair to first documented recurrence. The primary outcome was hernia recurrence. Secondary outcomes included 30‑day postoperative complications (including SSI, SSO, reoperation, ileus, small bowel obstruction, and readmission), mesh infection, and time to hernia recurrence. Reoperation within 30 days included formal re‑exploration and management of postoperative complications but did not include delayed primary closure.

**Fig. 1:**
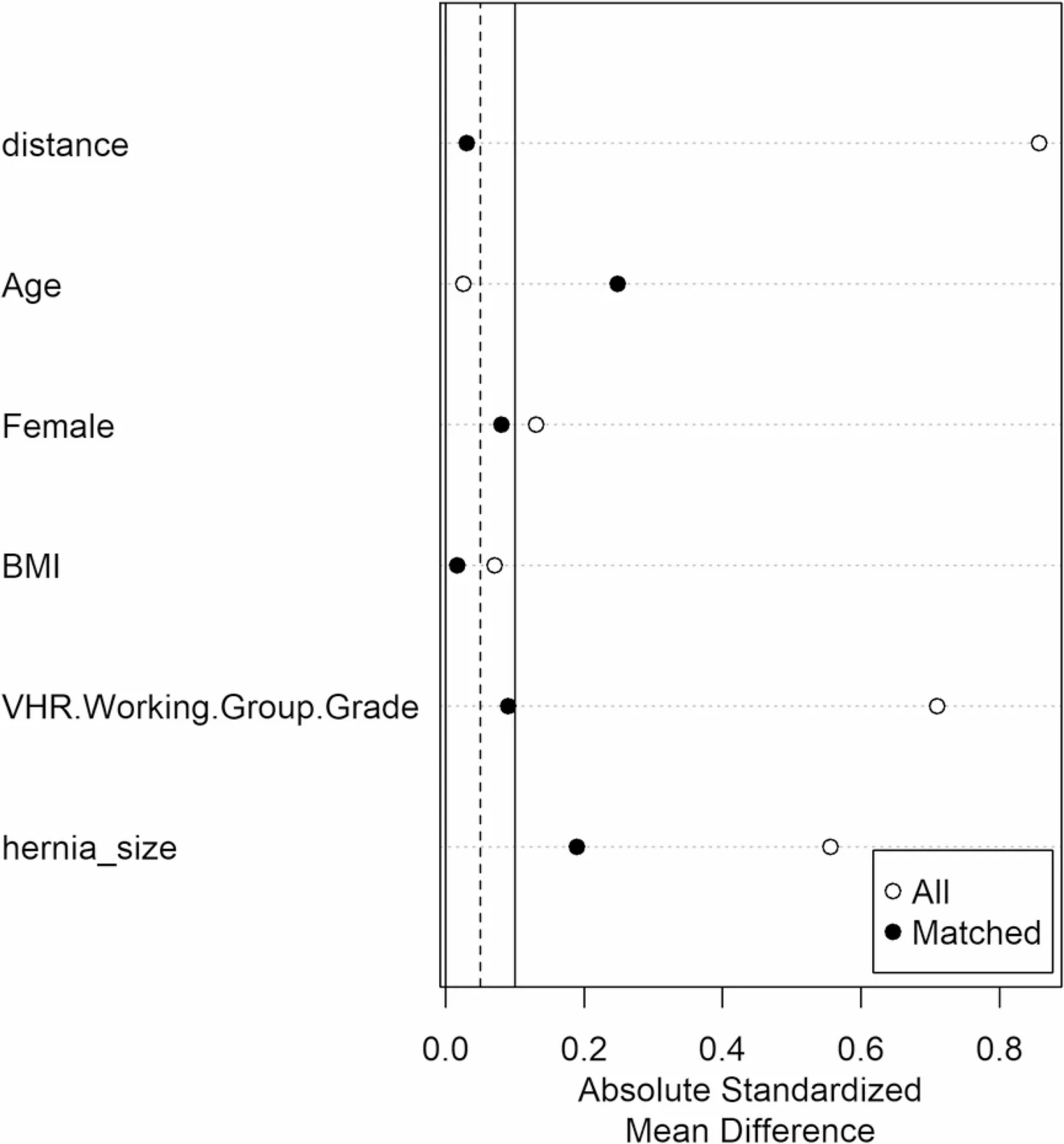
Demonstrates the overall distance between the two groups, before and after they were matched by age, sex, BMI, diabetes, ventral hernia working group and hernia size

Continuous variables were summarized as mean and standard deviation for normally distributed variables and median and interquartile range for non-normally distributed variables. Categorical variables were summarized as counts and percentages. Two-group comparisons were performed using Student’s t-test or Wilcoxon rank-sum test for continuous variables and chi-square test or Fisher’s exact test for categorical variables, as appropriate. Three group comparisons were performed using one-way ANOVA or Kruskal-Wallis tests for continuous variables and chi-square or Fisher’s exact tests for categorical variables. Patients treated with P4HB mesh were matched 1:1 to patients treated with permanent synthetic mesh using propensity scores derived from age, sex, body mass index, diabetes, CDC wound class, VHWG grade, and hernia size. Balance between groups after matching was assessed by comparing distributions of baseline variables (Fig. 1). Key comparative outcomes are now reported with effect estimates and 95% confidence intervals where feasible. Mesh selection was not randomized and based on surgeon judgment, considering the size of the defect, wound contamination, tissue quality, and patient-specific factors. All procedures were performed by fellowship-trained surgeons with anywhere from 5 to 15 years of independent practice experience.

Due to the limited number of biologic mesh cases (*n* = 22), they were excluded from the propensity-matched analysis. In a separate post hoc exploratory analysis, a matched cohort of patients who received P4HB versus biologic mesh was constructed using the same propensity score matching variables (age, sex, BMI, diabetes status, Centers for Disease Control and Prevention wound class, Ventral Hernia Working Group grade, and hernia size). Due to the limited biologic mesh sample size, only 8 matched pairs were achieved, and these results are presented in the supplemental materials for hypothesis-generating purposes only (Supplemental Table 1). Because this was a retrospective analysis of all eligible cases during the study period, no a priori sample size or power calculation was performed. This study was conducted and reported in accordance with the STROBE recommendations for observational studies. All statistical analyses were performed with R version 4.0.3 (R Foundation for Statistical Computing, Vienna, Austria) statistical software. All statistical tests were two-sided with statistical significance level set at p values < 0.05.

## Results

### Overall cohort

We started with 506 patients who underwent ventral hernia repair. Thirty-two patients with non-ventral hernias were excluded, and 71 additional patients were excluded due to missing mesh type information, leaving a final sample of 403 ventral hernia repairs for analysis. Forty-five received P4HB mesh, 22 received biologic mesh, and 336 received synthetic mesh. Age and sex distributions were similar across groups, with mean age of 59 years. BMI was higher in the biologic group (33.2 ± 6.0 kg/m²) compared with the P4HB (30.0 ± 7.0 kg/m²) and synthetic groups (29.4 ± 5.7 kg/m², overall *p* = 0.02) (Table 1). The distribution of specific mesh types is shown in Supplemental Table 2.

**Table 1:** Overall Descriptive Summary Statistics

	Bio-absorbable (Phasix) (*N* = 45)	Biologic (*N* = 22)	Synthetic (*N* = 336)	*p* value
Age				0.77^1^
N	45	22	336	
Mean (SD)	59.4 (15.0)	56.8 (13.1)	58.2 (14.6)	
Sex	22 (48.9%)	9 (50.0%)	124 (39.1%)	0.33^2^
Race				0.05^2^
White	40 (88.9%)	12 (66.7%)	245 (75.9%)	
Black	5 (11.1%)	2 (11.1%)	49 (15.2%)	
Other	0 (0.0%)	4 (22.2%)	29 (9.0%)	
Ethnicity	38 (86.4%)	12 (75.0%)	242 (79.1%)	0.47^2^
BMI				0.02^1^
N	44	18	316	
Mean (SD)	30.0 (7.0)	33.2 (6.0)	29.4 (5.7)	
Hypertension	23 (51.1%)	12 (54.5%)	184 (54.9%)	0.89^2^
Diabetes mellitus	9 (20.0%)	9 (40.9%)	60 (17.9%)	0.03^2^
COPD	2 (4.4%)	3 (13.6%)	25 (7.5%)	0.40^2^
Obstructive sleep apnea	6 (13.6%)	1 (5.9%)	42 (13.6%)	0.66^2^
Asthma	8 (18.2%)	0 (0.0%)	31 (10.1%)	0.10^2^
Inflammatory bowel disease	7 (15.6%)	3 (13.6%)	15 (4.5%)	< 0.01^2^
Hernia size				< 0.01^3^
N	37	19	238	
Median (Q1, Q3)	150.0 (81.0, 260.0)	28.0 (7.9, 277.5)	40.9 (9.0, 107.0)	
Mesh size				< 0.01^3^
N	45	21	302	
Median (Q1, Q3)	864.0 (500.0, 1050.0)	281.2 (80.0, 900.0)	162.0 (64.2, 470.2)	
Ventral Hernia Working Group Grade				< 0.01^2^
1	14 (31.8%)	2 (9.1%)	207 (63.1%)	
2	11 (25.0%)	7 (31.8%)	101 (30.8%)	
3	17 (38.6%)	12 (54.5%)	20 (6.1%)	
4	2 (4.5%)	1 (4.5%)	0 (0.0%)	
CDC Wound Status				< 0.01^2^
1	21 (46.7%)	9 (50.0%)	295 (91.6%)	
2	13 (28.9%)	7 (38.9%)	23 (7.1%)	
3	6 (13.3%)	1 (5.6%)	3 (0.9%)	
4	5 (11.1%)	1 (5.6%)	1 (0.3%)	
Operative time (minutes)				< 0.01^1^
N	34	14	282	
Mean (SD)	308.2 (167.9)	267.4 (182.6)	176.7 (100.7)	
Mesh infection	0 (0.0%)	1 (4.5%)	4 (1.2%)	0.29^2^
Hernia recurrence	3 (6.8%)	2 (9.1%)	14 (4.3%)	0.48^2^
Time until hernia recurrence (months)				0.83^3^
N	2	2	14	
Median (Q1, Q3)	30.0 (16.5, 43.5)	9.0 (6.5, 11.5)	7.0 (2.8, 13.0)	
30-day Surgical Site Infection(s)	1 (2.3%)	4 (19.0%)	18 (5.5%)	0.02^2^
30-day Surgical Site Occurrence(s)	5 (11.9%)	1 (8.3%)	36 (13.7%)	0.83^2^
30-day reoperation	4 (8.9%)	2 (9.1%)	15 (4.5%)	< 0.01^2^
30-day ileus	6 (13.3%)	1 (4.5%)	6 (1.8%)	< 0.01^2^
30-day SBO	3 (6.7%)	0 (0.0%)	1 (0.3%)	< 0.01^2^
30-day UTI	0 (0.0%)	0 (0.0%)	2 (0.6%)	0.82^2^

Across the three‑group comparison, most comorbidities were balanced. Diabetes mellitus was more frequent in the P4HB (20.0%) and biologic (40.9%) groups than in the synthetic group (17.9%; *p* = 0.03). Inflammatory bowel disease showed a similar pattern, occurring in 15.6% of P4HB and 13.6% of biologic patients versus 4.5% of synthetic mesh patients (*p* < 0.01). P4HB patients also had larger defects and implants. Median hernia size was 150.0 cm² (Q1–Q3 81.0–260.0) for P4HB, compared with 28.0 cm² (7.9–277.5) for biologic and 40.9 cm² (9.0–107.0) for synthetic mesh (*p* < 0.01). Median mesh size was 864.0 cm² (500.0–1050.0) in the P4HB group, versus 281.2 cm² (80.0–900.0) in the biologic group and 162.0 cm² (64.2–470.2) in the synthetic group (*p* < 0.01). P4HB and biologic groups also had higher VHWG grades and higher CDC wound classes than the synthetic group (both *p* < 0.01), indicating more complex and contaminated fields. Mean operative time was longest in the P4HB group (308.2 ± 167.9 min), followed by the biologic group (267.4 ± 182.6 min) and the synthetic group (176.7 ± 100.7 min; *p* < 0.01).

In the overall three-group analysis, mesh infection rates were low and comparable across groups. Hernia recurrence rates were also low, with a median time to recurrence of 30.0 months for P4HB, 9.0 months for biologic, and 7.0 months for synthetic (*p* = 0.83). P4HB was associated with higher 30-day reoperation (17.8% vs. 9.1% biologic vs. 4.5% synthetic, *p* < 0.01), ileus (13.3% vs. 4.5% biologic vs. 1.8% synthetic, *p* < 0.01), and small bowel obstruction (6.7% vs. 0% biologic vs. 0.3% synthetic, *p* < 0.01). Thirty-day surgical site infections were highest in biologic mesh (19% vs. 2.3% P4HB vs. 5.5% synthetic, *p* < 0.02), as well as 30-day readmission (22.7% vs. 13.3% P4HB vs. 7.5% of synthetic, *p* = 0.03).

In the direct, unmatched comparison limited to P4HB (*N* = 45) and synthetic mesh (*N* = 336), age, sex, BMI, and most comorbidities remained similar between groups (Table 2). Inflammatory bowel disease was more common among P4HB patients (15.6% vs. 4.5%, *p* < 0.01), whereas other comorbidities such as hypertension, diabetes, COPD, and obstructive sleep apnea did not differ significantly. P4HB patients continued to have larger repairs than synthetic mesh patients. Median hernia size was 150.0 cm² (81.0–260.0) in the P4HB group versus 40.9 cm² (9.0–107.0) in the synthetic group (*p* < 0.01), and median mesh size was 864.0 cm² (500.0–1050.0) versus 162.0 cm² (64.2–470.2), respectively (*p* < 0.01).

**Table 2:** Summary Statistics - Bio-absorbable vs. synthetic

	Bio-absorbable (Phasix) (*N* = 45)	Synthetic (*N* = 336)	*p* value
Age			0.60^1^
N	45	336	
Mean (SD)	59.4 (15.0)	58.2 (14.6)	
Sex	22 (48.9%)	124 (39.1%)	0.21^2^
Race			0.07^2^
White	40 (88.9%)	245 (75.9%)	
Black	5 (11.1%)	49 (15.2%)	
Other	0 (0.0%)	29 (9.0%)	
Ethnicity	38 (86.4%)	242 (79.1%)	0.26^2^
BMI			0.52^1^
N	44	316	
Mean (SD)	30.0 (7.0)	29.4 (5.7)	
Hypertension	23 (51.1%)	184 (54.9%)	0.63^2^
Diabetes mellitus	9 (20.0%)	60 (17.9%)	0.73^2^
COPD	2 (4.4%)	25 (7.5%)	0.46^2^
Obstructive sleep apnea	6 (13.6%)	42 (13.6%)	0.99^2^
Asthma	8 (18.2%)	31 (10.1%)	0.11^2^
Inflammatory bowel disease	7 (15.6%)	15 (4.5%)	< 0.01^2^
Cancer history	14 (31.1%)	68 (20.3%)	0.10^2^
Abdominal wall mets	1 (2.9%)	1 (0.4%)	0.08^2^
End Stage Renal Disease	2 (4.5%)	12 (3.6%)	0.75^2^
History of thrombosis	2 (4.4%)	19 (5.8%)	0.70^2^
Type of thrombosis			0.67^2^
1: PE	0 (0.0%)	4 (23.5%)	
2: DVT	2 (100.0%)	12 (70.6%)	
3: Both	0 (0.0%)	1 (5.9%)	
Hernia size			< 0.01^3^
N	37	238	
Median (Q1, Q3)	150.0 (81.0, 260.0)	40.9 (9.0, 107.0)	
Mesh size			< 0.01^3^
N	45	302	
Median (Q1, Q3)	864.0 (500.0, 1050.0)	162.0 (64.2, 470.2)	
Ventral Hernia Working Group Grade			< 0.01^2^
1	14 (31.8%)	207 (63.1%)	
2	11 (25.0%)	101 (30.8%)	
3	17 (38.6%)	20 (6.1%)	
4	2 (4.5%)	0 (0.0%)	
CDC Wound Status			< 0.01^2^
1	21 (46.7%)	295 (91.6%)	
2	13 (28.9%)	23 (7.1%)	
3	6 (13.3%)	3 (0.9%)	
4	5 (11.1%)	1 (0.3%)	
Operative time (minutes)			< 0.01^1^
N	34	282	
Mean (SD)	308.2 (167.9)	176.7 (100.7)	
Mesh infection	0 (0.0%)	4 (1.2%)	0.46^2^
Hernia recurrence	3 (6.8%)	14 (4.3%)	0.45^2^
Time until hernia recurrence (months)			0.53^3^
N	2	14	
Median (Q1, Q3)	30.0 (16.5, 43.5)	7.0 (2.8, 13.0)	
30-day Surgical Site Infection(s)	1 (2.3%)	18 (5.5%)	0.36^2^
30-day Surgical Site Occurrence(s)	5 (11.9%)	36 (13.7%)	0.75^2^
30-day reoperation	4 (8.9%)	15 (4.5%)	< 0.01^2^
30-day ileus	6 (13.3%)	6 (1.8%)	< 0.01^2^
30-day SBO	3 (6.7%)	1 (0.3%)	< 0.01^2^
30-day UTI	0 (0.0%)	2 (0.6%)	0.60^2^
30-day renal failure	2 (4.4%)	2 (0.6%)	0.02^2^
30-day PE	1 (2.2%)	4 (1.2%)	0.57^2^
30-day DVT	0 (0.0%)	2 (0.6%)	0.60^2^
30-day pneumonia	2 (4.4%)	3 (0.9%)	0.05^2^
30-day respiratory failure	0 (0.0%)	3 (0.9%)	0.52^2^
30-day respiratory failure requiring reintubation	0 (0.0%)	2 (2.7%)	0.77^2^
30-day unplanned transfer to ICU	3 (6.7%)	7 (2.1%)	0.07^2^
30-day mortality	0 (0.0%)	1 (0.3%)	0.71^2^
30-day other complications	6 (13.6%)	18 (5.4%)	0.04^2^
30-day readmission	6 (13.3%)	25 (7.5%)	0.18^2^

*P*-value based on: ^1^Linear Model ANOVA;^2^Pearson’s Chi-squared test;^3^Chi-squared test for given probabilities;^4^Kruskal-Wallis rank sum test

Distribution of VHWG grades and CDC wound class showed significantly more complex and contaminated cases in the P4HB group compared to the synthetic group (*p* < 0.01). Mean operative time (in minutes) remained significantly longer in the P4HB group (308.2 ± 167.9) compared with the synthetic group (176.7 ± 100.7, *p* < 0.01). Mesh infection (*p* = 0.46), hernia recurrence (*p* = 0.45), median time to recurrence (*p* = 0.53), 30-day SSI (*p* = 0.36), and SSO (*p* = 0.75) were comparable between groups. Thirty-day reoperation was more frequent in P4HB patients (8.9%) than in synthetic mesh patients (4.5%, *p* < 0.01). In the eight P4HB patients who underwent reoperation within 30 days, four were scheduled delayed primary closures and were therefore not included, two were for enterotomies (one bowel and one ureteral), and two were for postoperative infection. Of the 15 synthetic reoperations, 13 were due to infection, with one for choledocholithiasis, and one for a pelvic fluid collection. Thirty-day ileus had higher rates in P4HB patients (13.3% vs. 1.8%, *p* < 0.01), small bowel obstruction (6.7% vs. 0.3%, *p* < 0.01), renal failure (4.4% vs. 0.6%, *p* = 0.02), pneumonia (4.4% vs. 0.9%, *p* = 0.05), and other unspecified complications (13.6% vs. 5.4%, *p* = 0.04). Rates of 30-day unplanned ICU transfer, mortality, and readmission did not differ significantly between P4HB and synthetic mesh patients.

## Propensity score–matched cohort

After propensity score matching on age, sex, BMI, diabetes status, CDC wound class, VHWG grade, and hernia size, 25 patients who received P4HB mesh were matched to 25 patients who received synthetic mesh **(**Table 3**)**. Balancing of these variables is demonstrated in Fig. 1. Operative time was similar between groups (251.2 ± 84.7 vs. 262.9 ± 119.2 min, *p* = 0.73). Mesh infection occurred in 0% of P4HB patients and 8.0% of synthetic mesh patients (*p* = 0.15), and hernia recurrence was low and not significantly different (4.0% P4HB vs. 8.0% synthetic, *p* = 0.55). Thirty‑day surgical site infection was less frequent in the P4HB group (0% vs. 16.7%, *p* = 0.04), and surgical site occurrence was also lower (4.2% vs. 33.3%, *p* = 0.01). Other 30‑day outcomes, including reoperation, ileus, small bowel obstruction, renal failure, unplanned ICU transfer, mortality, and readmission, were not statistically different between P4HB and synthetic mesh patients.

**Table 3:** Matched by age, sex, BMI, diabetes, CDC wound status, VHWG grade, and hernia size

	Bio-absorbable (Phasix) (*N* = 25)	Synthetic (*N* = 25)	*p* value
Operative time (minutes)	251.2 (84.7)	262.9 (119.2)	0.73^1^--
Mesh subtypes			
Ultrapro Advanced	0 (0.0%)	10 (40.0%)	
Prolene Soft	0 (0.0%)	5 (20.0%)	
Phasix	25 (100.0%)	0 (0.0%)	
Bard Soft	0 (0.0%)	4 (16.0%)	
Ventralight ST	0 (0.0%)	4 (16.0%)	
Prolene polypropylene	0 (0.0%)	2 (8.0%)	
Mesh infection	0 (0.0%)	2 (8.0%)	0.15^2^
Hernia recurrence	1 (4.0%)	2 (8.0%)	0.55^2^
30-day Surgical Site Infection(s)	0 (0.0%)	4 (16.7%)	0.04^2^
30-day Surgical Site Occurrence(s)	1 (4.2%)	7 (33.3%)	0.01^2^
30-day reoperation	2 (8.0%)	3 (12.0%)	0.64^2^
30-day ileus	3 (12.0%)	1 (4.0%)	0.30^2^
30-day SBO	2 (8.0%)	0 (0.0%)	0.15^2^
30-day UTI	0 (0.0%)	0 (0.0%)	
30-day renal failure	1 (4.0%)	1 (4.0%)	1.00^2^
30-day PE	1 (4.0%)	0 (0.0%)	0.31^2^
30-day DVT	0 (0.0%)	1 (4.0%)	0.31^2^
30-day pneumonia	0 (0.0%)	0 (0.0%)	
30-day respiratory failure	0 (0.0%)	1 (4.0%)	0.31^2^
30-day respiratory failure requiring reintubation	0 (0.0%)	1 (4.0%)	
30-day unplanned transfer to ICU	2 (8.0%)	2 (8.0%)	1.00^2^
30-day mortality	0 (0.0%)	1 (4.0%)	0.31^2^
30-day other complications	3 (12.5%)	1 (4.0%)	0.28^2^
30-day readmission	3 (12.0%)	1 (4.0%)	0.30^2^

## Discussion

In our institution’s unmatched cohort of ventral hernia repairs with mesh, P4HB mesh was preferentially used in patients with larger hernia defects, higher Ventral Hernia Working Group grades, and more contaminated CDC wound classes, indicating that biosynthetic mesh was reserved for more complex ventral hernia repairs rather than routine cases. This reflects trends documented in which biosynthetic meshes are generally selected for high‑risk or contaminated fields compared to synthetic meshes, introducing substantial selection bias. In this context, worse early morbidity observed in the unmatched P4HB cohort (particularly higher rates of reoperation and postoperative complications) likely reflects underlying case complexity rather than problems relating to the mesh itself, highlighting the importance of propensity score matching. After matching, the P4HB cohort demonstrated lower 30-day SSI and SSO, and similar short-term recurrence and mesh infection rates, supporting the hypothesis that the apparent disadvantage seen in unmatched analyses is due to underlying case complexity. To our knowledge, this is the first series to apply propensity score matching specifically to P4HB versus permanent synthetic mesh, for isolated comparison of mesh performance.

There are four important findings from our study. First, our institutional experience of P4HB mesh use in patients with more complex defects is consistent with literature showing biosynthetic meshes (like P4HB) are preferentially used in more complex or contaminated hernias. Multiple studies show that P4HB mesh is specifically utilized in contaminated and complex ventral hernia repairs. A 2024 multicenter European study of 236 P4HB cases found that VHWG Grade 3 repairs were most frequent (49.1%), followed by Grade 2 (42.3%), with only 8.4% in Grade 1 cases, demonstrating clear preferential selection for more contaminated fields [8]. A French multicenter study acknowledges this selection bias, but is unable to address it, noting that 79% of P4HB cases were WHWG grade 3, with only 21% grade 2 [14]. A 2025 study specifically examined P4HB use in CDC class 3 or 4 contamination with high-risk comorbidities, reporting acceptable recurrence rates of 16% after emergency and 20% after elective procedures at 41.8 months follow-up [15]. Studies focusing on contaminated repairs report P4HB outcomes in CDC class II-IV wounds. Data from our unmatched cohort mirror these findings in published literature. P4HB at our institution was used in patients with substantially larger median hernia defects (150.0 cm² vs. 40.9 cm²) and mesh sizes (864.0 cm² vs. 162.0 cm²), with higher proportions of VHWG grade 3–4 and CDC class 3–4 wounds and longer operative times compared to synthetic mesh, confirming that P4HB was similarly reserved for more complex, contaminated repairs. Notably, population-level analysis of data found that although biologic or biosynthetic mesh was more likely to be used in urgent repairs and contaminated fields, nearly 90% of biologic and biosynthetic mesh use actually occurred in clean operative fields, suggesting inappropriate use patterns and significant practice variation [9]. Taken together, these external series and our institutional experience support the conclusion that the apparent excess morbidity seen in unmatched P4HB cohorts primarily reflects systematic selection of higher-risk cases rather than inferiority of the mesh itself.

Second, in our propensity-matched cohort with a mean follow-up of 12-months, our primary outcome of hernia recurrence was low and similar between P4HB and permanent synthetic mesh. Despite reporting significant selection bias, Morrison et al. showed comparable 5-year recurrence-free survival rates of approximately 72% for both P4HB and synthetic mesh types [16]. A recent 2025 matched analysis by Khan et al. specifically addressed this bias by using optimal full matching for P4HB versus synthetic mesh in clean wounds, finding comparable 3-year recurrence rates (3.3% vs. 7.0%, *p* = 0.4) despite the biosynthetic group having larger defects (220 cm² vs. 141 cm², *p* = 0.010), which are similar to the findings in our matched cohort [17]. In our matched cohort, hernia recurrence remained low in both groups at a mean of 12 months (4.0% with P4HB vs. 8.0% with synthetic mesh, *p* = 0.55), aligning more closely with synthetic mesh performance than with the higher recurrence rates reported in some single‑arm P4HB series. However, given our modest sample size and 12-month average follow-up, our data cannot establish long-term equivalence in recurrence but instead supports that short-term recurrence with P4HB mesh is comparable to synthetic mesh.

Only two previous studies have attempted propensity score matching to address this bias. Rodriguez-Quintero et al. compared absorbable synthetic mesh (including P4HB) to permanent mesh in CDC class II-III repairs, finding equivalent 30-day surgical site occurrences (15% vs. 16%) but significantly higher recurrence at 12 months with absorbable mesh (40% vs. 23%), which differs from our findings [18]. More recently, Patel et al. demonstrated that absorbable mesh (synthetic and biologic combined) had higher recurrence risk than permanent mesh across all wound classes [19]. While our unmatched data reflects this selection bias, our propensity-matched analysis suggests that recurrence rates with P4HB mesh are comparable to those with permanent synthetic mesh when wound complexity is balanced. These findings can also be interpreted to suggest that permanent synthetic mesh may perform at least as well as long‑acting resorbable mesh in complex hernia repair, given the similar recurrence rates and mesh infection rates observed after matching. In our study, the main differences after matching were limited to early wound outcomes rather than the primary durability outcome of recurrence. Accordingly, these data support P4HB as a selective option in certain complex settings, rather than a clearly superior substitute for permanent synthetic mesh.

A third notable finding relates to the timeline of hernia recurrence. In our unmatched cohort, median time to recurrence was 30.0 months for P4HB, 9.0 months for biologic mesh, and 7.0 months for synthetic mesh, with synthetic mesh recurrences occurring across the entire follow‑up window whereas the limited P4HB recurrences clustered between 18 and 36 months. Considering that P4HB is designed to resorb over approximately 18–24 months, late failures may emerge after complete resorption [20]. However, our pooled data and prior series with follow‑up up to 60 months have not demonstrated a clear divergence in recurrence risk compared with synthetic mesh [6, 7]. Large prospective multi-center studies have reported recurrence rates of 14–22% at roughly 42–60 months in predominantly VHWG grade 3 patients [7, 8]. A 2024 meta-analysis of 21 P4HB studies reported pooled recurrence of 9% overall with follow-up as long as 62 months, though these were predominantly single-arm cohorts without direct synthetic mesh comparisons [6]. A recent survival-kinetics analysis suggested that long-acting resorbable meshes (including P4HB) may have lower 5-year hernia recurrence rates compared to synthetic and biologic meshes, but these findings are exploratory [21]. However, it should be noted that not all long-term series of P4HB mesh in complex or contaminated fields have reported low recurrence rates and wound events. In a multicenter cohort of 108 patients with VHWG grades 3–4 undergoing incisional hernia repair with P4HB mesh in contaminated fields, Layer et al. observed a 22.2% clinical recurrence rate at a median follow-up of 41 months, with SSOs in 36.1% and SSIs in 24.1% [22]. Claessen et al. similarly reported approximately 10% recurrence in 71 patients treated with mid- and long-term degradable biosynthetic meshes (including P4HB) at a median follow-up of about 20 months, highlighting that durability may vary across indications and mesh locations and is not uniformly superior to permanent synthetic mesh [23]. Together with our institutional data, these findings suggest that long‑acting resorbable P4HB mesh may maintain acceptable durability over several years, although definitive comparisons to permanent synthetic mesh still require larger cohorts and longer‑term follow-up data.

The fourth finding from our study relates to short-term wound complications and infection profile. In our unmatched cohort, P4HB patients experienced significantly higher rates of early postoperative morbidity, including reoperation (17.8% vs. 4.5%), ileus (13.3% vs. 1.8%), small bowel obstruction (6.7% vs. 0.3%), and renal failure (4.4% vs. 0.6%) compared to synthetic mesh patients. However, these differences likely reflect the substantially greater case complexity in the P4HB group, characterized by larger hernia defects (median 150 cm² vs. 41 cm²), higher VHWG grades, more contaminated CDC wound classes, and nearly double the operative time (308 vs. 177 min). This interpretation is strongly supported by the propensity-matched cohort as most of these early complications became statistically similar between groups. In the propensity‑matched cohort, P4HB mesh was associated with significantly lower 30‑day surgical site infection (0% vs. 16.7%, *p* = 0.04) and surgical site occurrence (4.2% vs. 33.3%, *p* = 0.01) compared to synthetic mesh, while other early outcomes including reoperation (14.7% vs. 11.8%, *p* = 0.72) and readmission (5.9% vs. 8.8%, *p* = 0.64) were comparable. Furthermore, the rate of scheduled delayed primary closure was high in the P4HB group (50%), and delayed primary closure has been shown to reduce wound complications and improve recurrence rates, which may also contribute to the favorable wound outcomes observed in both the matched and unmatched cohorts [24]. The unmatched findings in the P4HB cohort showing increased 30-day reoperation rates are likely due to its use in more contaminated fields. These findings are consistent with the theoretical expectation that resorbable mesh may reduce infection risk, while existing literature on P4HB infection outcomes has been mixed. Importantly, large propensity-matched studies comparing absorbable to permanent mesh in contaminated fields have generally found equivalent SSI rates (12–14% for both mesh types), challenging the idea that absorbable mesh reduces infection risk [18, 25]. Our data support the consideration of P4HB mesh as a potential option when there is concern for early wound infection, while recognizing that consistent infection benefits over synthetic mesh have not been established.

Our findings should be interpreted given several limitations. First, we conducted a retrospective, single-institution design that is susceptible to selection and information bias, despite the use of a prospectively maintained database and standardized follow‑up protocols. Second, propensity score matching balanced key baseline variables between the P4HB and synthetic groups but cannot account for confounding from other unmeasured factors. Third, the sample size of the matched cohort was modest, and our follow‑up duration of 12 months is shorter than the expected 18–24-month resorption period of P4HB, limiting our ability to fully assess long‑term durability and mesh‑related complications. Fourth, this analysis combines a range of ventral hernia types and operative approaches, which may limit generalizability to more homogeneous populations. Fifth, our findings may not be directly applicable to other biosynthetic or biologic meshes, as they focus specifically on P4HB mesh (a type of biosynthetic mesh, but not representative of all biosynthetic meshes) compared with a variety of permanent synthetic meshes. Sixth, only 25 matched pairs were available for the primary comparative analysis, limiting precision and increasing the possibility of both type II error and unstable estimates for uncommon outcomes. Seventh, although cultures were obtained in patients with suspected mesh infection, we did not perform a detailed organism‑level analysis for all patients, particularly with respect to pre‑existing versus postoperative infections, which limits our ability to correlate specific microbiologic patterns with mesh type and outcomes. Finally, a post hoc matched analysis of P4HB versus biologic mesh showed no statistically significant differences in 30-day surgical site infection, surgical site occurrence, or readmission, underscoring that the biologic cohort was underpowered for definitive comparative conclusions. Future research should focus on larger, multicenter prospective studies to confirm these results and refine patient selection for P4HB in complex ventral hernia repair.

## Conclusion

In this retrospective, single-center propensity-matched cohort, long-acting resorbable poly-4-hydroxybutyrate mesh demonstrates comparable recurrence and mesh infection rates with lower short-term wound morbidity after balancing key risk factors, suggesting bioabsorbable mesh to be a reasonable alternative to synthetic mesh in selected complex or contaminated ventral hernia repairs. Although larger multicenter studies with longer follow-up are needed before broader adoption, our findings suggest that the apparent excess morbidity that has been reported in unmatched poly-4-hydroxybutyrate cohorts is likely driven by its preferential use in higher-risk patients, rather than due to the material itself.

## Supplementary Information

Below is the link to the electronic supplementary material.

Supplementary Material 1
