# Supplementary material for: Comparative outcomes of bioabsorbable poly-4-hydroxybutyrate versus synthetic mesh in ventral hernia repair: A propensity-matched cohort study
Source: Hernia. 2026 Sep 16;30(1):349. doi: 10.1007/s10029-026-03848-8 (PMC13582305; doi:10.1007/s10029-026-03848-8)
Supplement: Supplementary file 1 [file 10029_2026_3848_MOESM1_ESM.docx]

| **Supplemental Table 1. Summary Statistics - Matched analysis: Phasix vs Biologic** | | | |
| --- | --- | --- | --- |
|  | Bio-absorbable (Phasix) (N=8) | Biologic (N=8) | p value |
| **Operative time (minutes)** |  |  | 0.91^1^ |
| N | 7 | 4 |  |
| Mean (SD) | 342.7 (149.1) | 328.5 (270.6) |  |
| **Mesh infection** | 0 (0.0%) | 0 (0.0%) |  |
| **Hernia recurrence** | 1 (12.5%) | 2 (25.0%) | 1.00^2^ |
| **30-day Surgical Site Infection(s)** | 0 (0.0%) | 3 (37.5%) | 0.20^2^ |
| **30-day Surgical Site Occurrence(s)** | 0 (0.0%) | 1 (20.0%) | 0.38^2^ |
| **30-day reoperation** | 2 (25.0%) | 2 (25.0%) | 1.00^2^ |
| **30-day ileus** | 2 (25.0%) | 0 (0.0%) | 0.47^2^ |
| **30-day SBO** | 1 (12.5%) | 0 (0.0%) | 1.00^2^ |
| **30-day UTI** | 0 (0.0%) | 0 (0.0%) |  |
| **30-day renal failure** | 0 (0.0%) | 0 (0.0%) |  |
| **30-day PE** | 0 (0.0%) | 0 (0.0%) |  |
| **30-day DVT** | 0 (0.0%) | 0 (0.0%) |  |
| **30-day pneumonia** | 1 (12.5%) | 0 (0.0%) | 1.00^2^ |
| **30-day respiratory failure** | 0 (0.0%) | 0 (0.0%) |  |
| **30-day respiratory failure requiring reintubation** | 0 (0.0%) | 0 (0.0%) |  |
| **30-day unplanned transfer to ICU** | 1 (12.5%) | 0 (0.0%) | 1.00^2^ |
| **30-day mortality** | 0 (0.0%) | 0 (0.0%) |  |
| **30-day other complications** | 0 (0.0%) | 0 (0.0%) |  |
| **30-day readmission** | 1 (12.5%) | 3 (37.5%) | 0.57^2^ |
